# The relationship between plasma free fatty acids, cognitive function and structural integrity of the brain in middle-aged healthy humans

**DOI:** 10.18632/aging.203573

**Published:** 2021-09-23

**Authors:** Markus Herrmann, Sebastian Simstich, Günter Fauler, Edith Hofer, Eva Fritz-Petrin, Wolfgang Herrmann, Reinhold Schmidt

**Affiliations:** 1 Clinical Institute of Medical and Chemical Laboratory Diagnostics, Medical University of Graz, Graz, Austria; 2 Clinical Division of Neurogeriatrics, Department of Neurology, Medical University of Graz, Graz, Austria; 3 Institute for Medical Informatics, Statistics and Documentation, Medical University of Graz, Graz, Austria; 4 Medical School, Saarland University, Homburg/Saar, Germany

**Keywords:** free fatty acids, NCI-GC-MS, PFBBr, cognitive function, memory

## Abstract

Background: The cerebral composition of ω-3 and ω-6 polyunsaturated fatty acids (PUFAs) is believed to influence cognitive function and structural damage of the aging brain. However, existing data is inconsistent.

Materials and Methods: This retrospective study explored the association between free plasma PUFA concentrations, cognitive function and brain structure atrophy in a well-characterized community-dwelling cohort of elderly individuals without stroke and dementia. Ten different fatty acids were analyzed in stored plasma samples from 391 non-demented elderly individuals by gas chromatography mass spectrometry. Neuropsychiatric tests capturing memory, executive function and visuopractical skills were performed in all participants. Brain atrophy was assessed by MRI in a subset of 167 individuals.

Results: Higher plasma concentrations of free ω-6 PUFAs (*p* = 0.042), and, in particular, linoleic acid (*p* = 0.01), were significantly associated with lower executive function. No significant association existed between ω-3 PUFA concentrations and cognitive functioning. The volume of the frontal lobes was inversely associated with ω-6 PUFAs, whereas ω-3 PUFAs were positively related with temporal lobe volumes. All associations did not withstand correction for multiple comparisons.

Conclusions: Our study suggests subtle effects of PUFA imbalances on cognition and brain structure. Yet the observed associations are weak and unlikely to be of clinical relevance. The brain regions that seem to be most sensitive to imbalances of ω-3 and ω-6 PUFAs are the frontal and temporal lobes.

## INTRODUCTION

Lipids account for nearly 60% of human brain tissue and the composition of fatty acids (FAs) seems to impact on brain integrity and function [1]. FAs are the elementary building blocks of triglycerides, phospholipids and cholesteryl esters. They function as energy sources and are important structural components of all cells. Polyunsaturated FAs (PUFAs) appear to play a key role in age-related cognitive decline and brain atrophy [2–4]. ω-3 and ω-6 PUFA include eicosapentaenoic acid (EPA, 20:5 ω-3), docosahexaenoic acid (DHA, 22:6 ω-3), linoleic acid (LA, 18:2 ω-6), γ-linolenic acid (GLA, 18:3 ω-6) and arachidonic acid (AA, 20:4 ω-6), respectively. While humans can synthesize saturated and monounsaturated FAs (MUFAs) they are not able to synthesize PUFA, such as GLA and LA or DHA and EPA, due to the absence of the conversion enzyme ω-3-desaturase [5].

In the brain, DHA is the most abundant PUFA species [6] accounting for more than 90% of all ω-3 PUFAs and for approximately 10–20% of all lipid species [7]. It is mainly incorporated in phosphatidylethanolamine, phosphatidylserine and in smaller amounts in phosphatidylcholine [8] at synaptic terminals, mitochondria and endoplasmic reticula. DHA is able to modulate cellular features and physiological processes, such as membrane fluidity, release of neurotransmitters, gene expression, myelination, neuroinflammation and neuronal growth [9]. The brain´s supply with PUFAs relies on their transfer across the blood brain barrier (BBB) by passive diffusion and specific transport mechanisms [10]. In blood, PUFAs circulate in their unbound form, bound to albumin [10] or esterified in triacylglycerols, phospholipids, cholesteryl esters or phosphatidylcholine [11]. Unbound free FAs (u-FFAs) are well correlated with total free FAs (t-FFAs), which include all non-esterified FAs. t-FFAs represent the FA fraction that can cross the BBB by passive diffusion and is readily available for metabolism [12]. This non-saturable process is regulated by plasma albumin so that plasma t-FFAs equilibrate to about 90% of maximal brain uptake within 15 min [13], and the higher the degree of unsaturation, the more rapid is the uptake [11].

The cerebral FA composition has been reported to influence cognitive function and structural damage of the aging brain [14]. In particular, FA saturation appears to be a critical determinant of AD risk [15, 16]. Cerebral FFAs have been related to an accelerated cognitive decline of dementia patients [2–4]. *In-vitro* experiments have demonstrated that FAs stimulate the assembly of amyloid β (Aβ) and tau filaments [17]. The proportion between ω-3 and ω-6 FA is thought to play a crucial pathogenic role in this context [18]. In line with this assumption, a diet rich in ω-3 FA, and/or low in ω-6 FA was found to lower the incidence of AD and other degenerative brain disorders [3, 19]. Other studies have measured plasma FAs in an attempt to estimate the risk of cognitive impairment and dementia that is attributable to imbalances in ω-3 and ω-6 FA composition [20–24]. The concept that ω-3 FAs protect against cognitive dysfunction and neurodegeneration, whereas ω-6 FA have detrimental effects, is supported by some investigations [2, 22–29] while others opposed this view [30–33]. Yagi et al. showed a positive correlation between EPA and the EPA/AA-ratio with performance on the Minimal Mental State Examination (MMSE) in Japanese coronary artery disease patients [24]. High plasma EPA concentrations have also been associated with better performance on specific cognitive functions, such as visual working memory [28], and were found to decrease the risk of dementia in general [25, 27]. The opposite was described for high plasma ω-6 PUFA concentrations [26] and unfavorable ratios between AA/DHA as well as ω-6/ω-3 PUFAs, which related to increased risk of dementia [25, 27, 29] and the progression of white matter lesions [23, 29]. These reports are contrasted by studies that failed to link ω-3 PUFA concentrations with cognitive decline, dementia or AD [30–32]. Moreover, a Cochrane Collaboration meta-analysis showed no benefit of ω-3 PUFA supplementation on cognitive function in cognitively healthy older people [33].

Existing studies are mainly limited by the measurement of total plasma FAs rather than the metabolically active t-FFAs. However, plasma t-FFA profiles may provide additional information about lipid metabolism and disease risk [34]. We here extend previous work by studying the association between plasma t-FFAs, cognitive function and brain structure in a well-characterized community-dwelling cohort of elderly individuals without stroke and dementia. The cognitive test battery was extensive, demanding and assessing different cognitive domains, and brain MRI was evaluated quantitatively with measurements of structural and microstructural brain alterations.

## MATERIALS AND METHODS

### Study design

Stored EDTA plasma samples from 391 participants of the Austrian Stroke Prevention Study (ASPS) were used for t-FFA measurement as described below. Cognitive function was assessed with a comprehensive battery of neuropsychiatric tests capturing memory, executive function and visuopractical skills. Furthermore, structural neurodegenerative and vascular changes were analyzed in 167 individuals who underwent brain MRI on the day of cognitive testing.

Results were used to explore associations between FFAs, cognitive function and structural markers of neurodegeneration. Statistical analyses included only results from participants with a complete set of biochemical and cognitive test results who did not supplement fatty acids. Participants with a family history of dementia were excluded from the statistical analyses (*n* = 23). Written informed consent was obtained from all participants and the study was approved by the ethics committee of the Medical University of Graz, Austria.

### Participants

The study population consisted of community-dwelling participants from the Austrian Stroke Prevention Study (ASPS) and the Austrian Stroke Prevention Family Study (ASPS-Fam). All participants had an unremarkable neurological status and no history of stroke and dementia. ASPS is a prospective single-center study examining the effects of vascular risk factors on brain structure and function [35, 36]. Randomly selected individuals from the community register of the city of Graz, Austria, were enrolled. ASPS-Fam is an extension of ASPS with a similar study protocol consisting of participants of ASPS and their first-grade relatives [37, 38].

Common risk factors assessed in all participants included arterial hypertension, diabetes mellitus, atrial fibrillation, hypercholesterolemia and hypertriglyceridemia. Hypertension was defined as history of hypertension or systolic blood pressure over 140 mmHg or a diastolic blood pressure over 90 mmHg [39] and current use of antihypertensive agents. Subjects were classified as diabetic on the basis of a documented history of diabetes, use of anti-diabetics or a fasting blood glucose level above 126 mg/dl (7.0 mmol/L) at the time of examination [40]. The presence of atrial fibrillation was confirmed by an electrocardiogram obtained during the study visit. Presence of hypercholesterolemia was confirmed if a participant had a history of hypercholesterolemia, was treated for hypercholesterolemia at the time of examination or if the total or LDL cholesterol was higher than 200 mg/dl or 130 mg/dl respectively. Hypertriglyceridemia was defined as history of hypertriglyceridemia or treatment for hypertriglyceridemia or triglyceride levels of at least 200 mg/dl.

### Measurement of free fatty acids

Ten different FFAs were analyzed by a modified gas chromatography mass spectrometry (GC-MS) method with liquid-liquid sample extraction [41, 42]. Briefly, 50 μL of plasma were mixed with 600 μL of deionized water, 500 μL of 0.5 N methanolic hydrochloric acid and 4 mL Methyl tert-butyl ether (MTBE) in a 10 mL silanized glass tube. Furthermore, internal standard solutions (IS) containing 1 nmol of C18d35 and C21:0 were added. This mixture was vigorously vortexed for 30 s and subsequently centrifuged for 2 min at 1500 g. The upper organic layer was carefully transferred in a 10 mL silanized glass tube. The remaining aqueous layer was extracted again with 4 mL MTBE. After centrifugation, the MTBE phase of the second extraction was added to the first one. The combined extracts in the glass tube were dried under a gentle stream of nitrogen. In the next step, samples were derivatized using a modified protocol from Quehenberger et al. (2011). The dried extracts were resuspended and derivatized in 25 μL of 1% diisopropylethylamine in acetonitrile (DIPEA) and 25 μL of 10% pentafluorobenzyl bromide in acetonitrile (PFBBr) followed by 20 min of incubation at room temperature. Then, solvents were removed by evaporation under a gentle stream of nitrogen. The residues were again resuspended in 100 μL of pure methanol, vigorously vortexed for 10 s and then dried under a gentle stream of nitrogen. In the last step, the dried samples were dissolved in 200 μL of hexane, vigorously vortexed for 10 s and carefully transferred to an autosampler glass vial and placed into the autosampler of the GC-MS instrument.

For analysis a Trace GC Ultra-DSQ II GC-MS (Thermo Scientific) equipped with a 30 m TR-FAME column (30 m, 0,25 mm, 0,25 μm, Thermo Scientific) was used. From the purified samples, 1 μl was injected into the instrument in split/splitless mode and immediately evaporated in the injector, which was set at a temperature of 250°C. Methane was used as carrier gas at a flow rate of 2 ml/min. Subsequently, the evaporated samples were transferred from the injector onto the analytical column, which was kept at 150°C in the oven. After 1 min of equilibration, temperature was raised at a rate of 20°C/min to 200°C, which was kept for 2 min. Subsequently, the temperature was further increased with a rate of 30°C/min to a final temperature of 300°C and kept for 5 min for column bake out. The eluate from the column was sent through the transfer line, set at 310°C, into the source, where it was ionized by negative chemical ionization (NCI) with methane at a source temperature of 250°C. Lastly, ions were introduced into a single quadrupole mass spectrometer where FFAs were detected in single ion monitoring mode (SIM). The SIM masses and the retention times for all analytes including the IS are listed in Table 1.

**Table 1:** SIM masses and retention time of the measured fatty acids.

**Fatty acid**	**Mass (m/z)**	**SIM mass (m-181) (m/z)**	**Internal Standard**	**Retention time (min)**
Palmitoleic acid (C16:0)	256.2	255.2	C18d35	6.16
Palmitolenic acid (C16:1)	254.2	253.2	C18d35	6.36
Stearic acid (C18:0)	284.3	283.3	C18d35	6.93
Oleic acid (C18:1)	282.3	281.3	C18d35	7.05
Linoleic acid (C18:2)	280.2	279.2	C18d35	7.26
Gamma linoleic acid (C18:3)	278.2	277.2	C18d35	7.37
Arachinoic acid (C20:0)	312.3	311.3	C21:0	7.51
Arachidonic acid (C20:4)	304.2	303.2	C21:0	7.92
Eicosapentaenoic acid (C20:5)	302.2	301.2	C21:0	8.11
Docosahexaenoic acid (C22:6)	328.2	327.2	C21:0	8.56
IS deuterated Stearic acid (C18d35)	319.7	318.7		6.78
IS Heneicosylic acid (C21:0)	326.3	325.3		7.76

Our method has been validated in accordance with FDA guidelines [43]. The measured concentrations of all FAs in our samples fell inside the validated analytical ranges for these compounds. Intra- and interassay imprecision were <15% for all compounds and across the entire analytical range.

### Neuropsychological testing

Cognitive function was assessed by a comprehensive battery of neuropsychological tests capturing memory, executive function and visuopractical skills. A detailed description of these tests has been published previously [35, 44–49]. Individual tests were summarized in composite measures of the cognitive domains memory, executive function and visuopractical skills. These summary measures were calculated by converting test results to z-scores based on the mean and standard deviation of the combined ASPS and ASPS-Fam sample, and by computing the average z-scores within each cognitive domain.

### Magnetic resonance imaging (MRI)

Study participants underwent MRI on a 3T whole-body MR system (TimTrio; Siemens Healthcare, Erlangen, Germany). The MRI subgroup included only those individuals in whom 3D T1 and FLAIR sequences were available.

Total, cortical and subcortical gray matter volume, hippocampal volume and lobar cortical volume, were computed from the T1 weighted MPRAGE images using FreeSurfer 5.3 [50, 51]. Based on the intensity of the voxels in the MRI image, the software automatically segments the brain into subcortical gray volumetric structures and cortical gray matter. Freesurfer divides the cerebral cortex into gyral based regions of interest and provides the cortical volume for each of these regions. Values of these regions were added up or averaged for volume, to obtain lobar measures. To correct for variations in individual head size, all measures were normalized for total intracranial volume.

Vascular lesions including white matter hyperintensities (WMH), silent non-lacunar and lacunar infarcts were assessed on FLAIR images by a blinded expert. WMH were outlined using a custom written IDL program (Exelis Visual Information Solutions, USA). Lesion areas were segmented by combined region growing and local thresholding following manual selection, as described earlier [52]. The total lesion volume (cubic millimeter) was calculated using the program FSLMATHS by multiplying the lesion area with the slice thickness and was normalized for head size. Due to a skewed distribution, the lesion load was logarithmically transformed. Lacunes were defined as focal lesions involving the basal ganglia, internal capsule, thalamus, brainstem, or the white matter, not exceeding a maximum diameter of 20 mm. We considered lesions with typical signal characteristics of infarcts following a typical vascular territory or located in a border zone between two vascular territories as non-lacunar infarcts.

To assess microstructural changes, we used the peak width of the skeletonized mean diffusivity (PSMD). This represents a new, robust, fully automated and easy-to-implement marker for cerebral small vessel disease based on diffusion tensor imaging, white matter tract skeletonization (as implemented in FSL-TBSS) and histogram analysis. This software package allows calculating PSMD from diffusion tensor imaging data

### Statistical analysis

Statistical analysis was performed with the R statistical software package version 3.6.1 [53]. We assessed normality of continuous variables by visual inspection and Shapiro-Wilk’s test. Normally distributed variables are reported as mean ± standard deviation (std) and non-normally distributed variables as median and interquartile range (IQR). We used linear mixed models to determine the association between FFAs and cognition as well as MRI. All analyses were adjusted for age, sex, hypertension, diabetes, atrial fibrillation, hypercholesterolemia and hypertriglyceridemia. Cognition analyses were additionally adjusted for years of education, and, as we pooled ASPS and ASPS-Fam data, for cohort to adjust for any undetected differences between the two studies. ASPS-Fam is a family study and therefore the family structure was included in our models as a random effect using the lmekin function of the R package coxme [54]. The degree of relationship between any two individuals in the study is represented by a kinship matrix, which was generated using the R package kinship2 [55]. The results of linear mixed model analyses are presented as regression coefficient (β), standard error of the regression coefficient (SE) and *p*-values (p). For all *p*-values within Tables 2–4 we applied false discovery rate (FDR) correction [56] to compensate for the number of tests in the Table.

**Table 2:** Associations between free ω3 and ω6 polyunsaturated fatty acids (PUFA) and cognitive function.

**Area of cognitive function**	**ω3-PUFA**					**ω6-PUFA**				
	**N**	**β**	**se**	***p***	**p_FDR_**	**N**	**β**	**se**	***p***	***p*_FDR_**
Executive function	368	–0.0094	0.0053	0.076	0.196	368	–0.0016	0.0006	0.007	0.042
Visuopractical skills	368	–0.0071	0.0083	0.395	0.593	368	–0.0016	0.0009	0.098	0.196
Memory	368	0.0012	0.0086	0.891	0.891	368	0.0003	0.0010	0.753	0.891

Abbreviations: N: sample size, beta: regression coefficient, se: standard error, *p*: *p*-value, *p*_FDR_: false discovery rate corrected *p*-value. Linear mixed model adjusted for age, sex, hypertension, diabetes, arterial fibrillation, hypercholesterolemia, hypertriglyceridemia and education.

**Table 3:** Significant associations between individual free plasma ω6 polyunsaturated fatty acids and executive function.

**Area of cognitive function**	**Executive function**				
	**N**	**β**	**se**	***p***	***p*_FDR_**
Linoleic Acid	368	–0.002	0.0007	0.01	0.02
Arachidonic Acid	368	–0.008	0.0036	0.03	0.03

Abbreviations: N: sample size, beta: regression coefficient, se: standard error, *p*: *p*-value, *p*_FDR_: false discovery rate corrected *p*-value. Linear mixed model adjusted for age, sex, hypertension, diabetes, arterial fibrillation, hypercholesterolemia and hypertriglyceridemia.

**Table 4:** Associations between ω3 and ω6 polyunsaturated fatty acids (PUFA) and MRI-derived morphologic features of cognitive impairment and dementia.

**Area of cognitive function**	**ω3-PUFA**					**ω6-PUFA**				
	**N**	**β**	**se**	***p***	***p*_FDR_**	**N**	**β**	**se**	***p***	***p*_FDR_**
Total gray volume	146	3.51E-04	3.71E-04	0.344	0.917	146	–7.93E-05	5.58E-05	0.155	0.620
Hippocampus volume	146	1.68E-06	4.96E-06	0.735	0.924	146	1.25E-07	7.21E-07	0.862	0.924
Frontal lobe volume	146	1.31E-05	1.23E-04	0.915	0.924	146	–4.61E-05	1.81E-05	0.011	0.136
Temporal lobe volume	146	1.59E-04	6.65E-05	0.017	0.136	146	–3.98E-06	1.02E-05	0.696	0.924
Parietal lobe volume	146	5.23E-05	8.49E-05	0.538	0.924	146	–1.94E-05	1.27E-05	0.127	0.620
Occipital lobe volume	146	–3.94E-06	4.11E-05	0.924	0.924	146	–3.33E-06	6.15E-06	0.588	0.924
WMH volume	146	–3.44E-05	1.16E-04	0.766	0.924	146	–1.17E-05	1.72E-05	0.497	0.924
PSMD	68	2.91E-03	2.88E-03	0.313	0.917	68	6.27E-05	3.99E-04	0.875	0.924

Abbreviations: N: sample size, beta: regression coefficient, se: standard error, *p*: *p*-value, *p*_FDR_: false discovery rate corrected *p*-value; WMH: white matter hyperintensities; PSMD: peak width of skeletonized mean diffusivity. Linear mixed model adjusted for age, sex, hypertension, diabetes, arterial fibrillation, hypercholesterolemia and hypertriglyceridemia.

## RESULTS

### Descriptive statistics

Demographics, frequency of risk factors and laboratory findings are displayed in Table 5. The cohort included more females than males. Participants had a median age of 68 years. Hypercholesterolemia was the most prevalent vascular risk factor in the ASPS cohort affecting 321 (82.1%) individuals. Statins and other lipid lowering drugs were used by 15% of all subjects.

**Table 5:** Demographics and risk factors.

**Parameter**	**Result**
N	391
Females, N (%)	250 (63.9%)
Age (years), median (IQR)	68 (62–74)
Education (years), median (IQR)	10 (9–13)
BMI, median (IQR)	26.5 (24.1–29.1)
Hypertension, N (%)	276 (70.6%)
Diabetes, N (%)	46 (11.8%)
Atrial fibrillation, N (%)	24 (6.1%)
Statins, N (%)	53 (13.6%)
Other Lipid lowering medication	6 (1.5%)

The median total FFA concentration was 307.5 μmol/L with a relative even distribution of saturated (31%), monounsaturated (39%) and polyunsaturated (30%) FFA. Table 6 shows the plasma concentrations of all measured FFA species. The plasma concentrations of ω-6 PUFA species were substantially higher than those of ω-3 PUFA species with a median ω-6/ω-3 ratio of 11.06 (IQR: 8.01–16.06).

**Table 6:** Median (IQR) concentrations of total and individual free fatty acids.

***Groups of FFA***		
Total FFA	(μmol/L)	307.52 (243.65–415.86)
Saturated FFA	(μmol/L)	94.91 (72.87–133.89)
Monounsaturated FFA	(μmol/L)	118.37 (91.53–167.13)
Polyunsaturated FFA	(μmol/L)	93.42 (76.39–119.34)
***Individual species of saturated FFA***		
Palmitic Acid (C16:0)	(μmol/L)	59.26 (43.03–84.39)
	(% of total FFA)	19.81 (16.19–22.51)
Stearic Acid (C18:0)	median (IQR)	36.99 (27.62–49.02)
	(% of total FFA)	11.43 (9.75–13.33)
Arachinic Acid (C20:0)	median (IQR)	0.15 (0.09–0.23)
	(% of total FFA)	0.05 (0.03–0.07)
***Individual species of monounsaturated FFA***		
Palmitoleic Acid (C16:1)	median (IQR)	20.27 (13.24–30.54)
	(% of total FFA)	6.35 (4.35–8.18)
Oleic Acid (C18:1)	median (IQR)	94.25 (74.87–142.26)
	(% of total FFA)	31.52 (28.93–34.53)
***Individual species of polyunsaturated FFA***		
Linoleic Acid (C18:2)	median (IQR)	68.89 (55.23–93.11)
	(% of total FFA)	22.47 (20.88–24.45)
γ-Linolenic Acid (C18:3)	median (IQR)	0.84 (0.48–1.36)
	(% of total FFA)	0.28 (0.16–0.45)
Arachidonic Acid (C20:4)	median (IQR)	14.82 (11.38–19.42)
	(% of total FFA)	4.66 (3.1–6.14)
Eicosapentaenoic Acid(C20:5)	median (IQR)	1.66 (1.13–2.43)
	(% of total FFA)	0.52 (0.33–0.77)
Docosahexaenoic Acid (C22:6)	median (IQR)	5.98 (4.25–8.29)
	(% of total FFA)	1.93 (1.3–2.75)
***ω3 and ω6 polyunsaturated FFA species***		
ω3PUFA (C22:6 + C20:5)	median (IQR)	7.82 (5.63–11.01)
ω6 PUFA (C18:2 + C20:4)	median (IQR)	85.02 (70.14–109.53)
ω6/ω3 ratio	median (IQR)	11.06 (8.01–16.06)

### Association between plasma free PUFAs and cognitive test results

**Figure 1:**
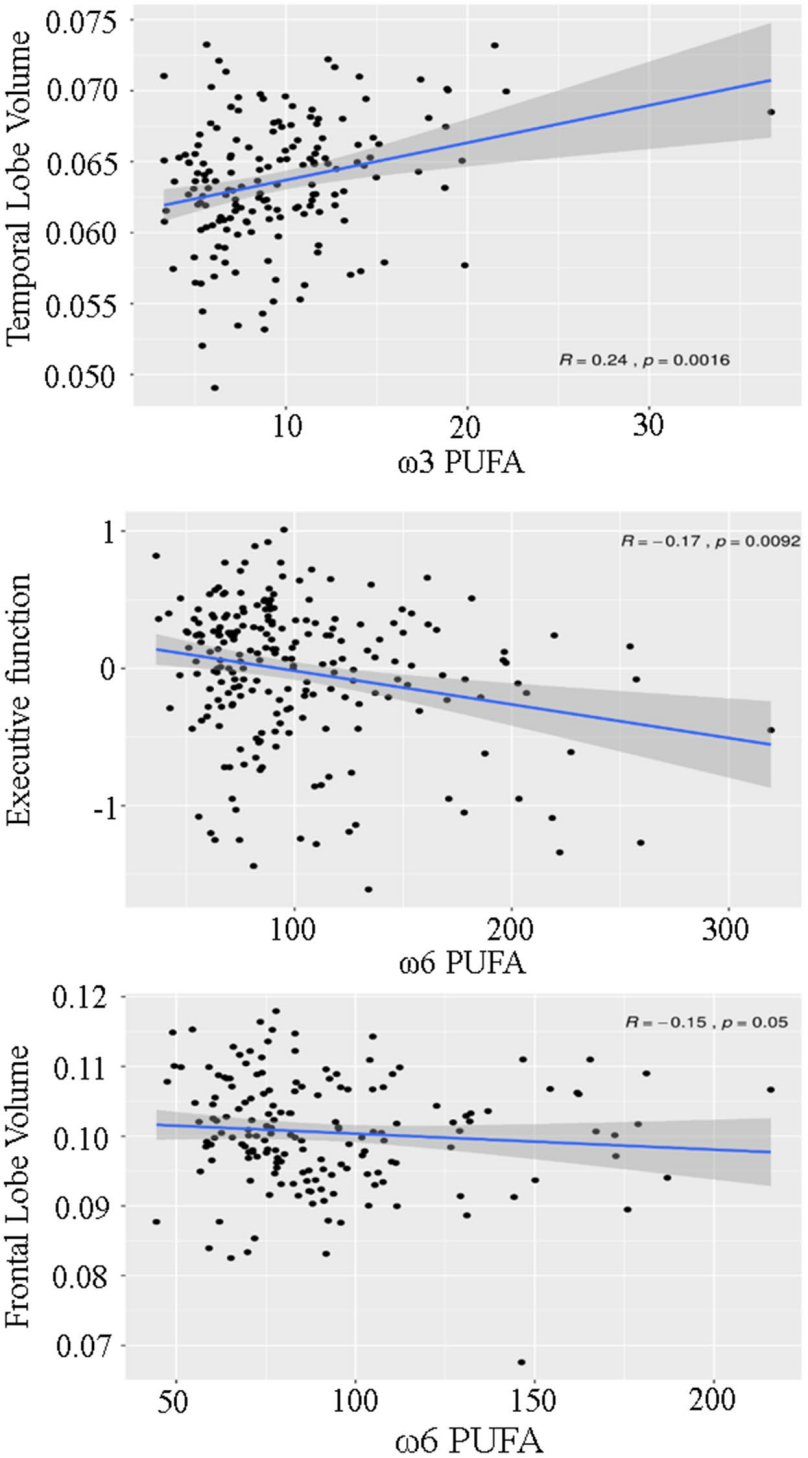
Scatterplot demonstrating the association between ω3 and ω6 polyunsaturated fatty acids (PUFA), temporal lobe volume, executive function and frontal lobe volume.

The associations between plasma PUFA concentrations and cognitive function were explored by using a linear mixed model adjusted for possible confounders (Table 2, Figure 1). This model showed a significant inverse relationship between ω-6 PUFA and executive function, which remained significant after correction for the expected false discovery rate due to multiple testing.

No significant association existed between free ω-6 PUFA concentrations, visuopractical skills and memory function. The link between ω-6 PUFA and executive function was primarily driven by the plasma concentration of linoleic acid (Table 3). Contrary to free ω-6 PUFA, the plasma concentrations of free ω-3 PUFA were not significantly related to any of the three cognitive domains.

### Association between plasma free PUFAs and MRI findings

A higher plasma ω-6 PUFA concentration was significantly associated with a lower volume of the frontal lobe (Table 4, Figure 1) while lower ω-3 PUFA concentrations related positively to the volume of the temporal lobe. Both associations lost significance when *p*-values were corrected for the expected false discovery rate due to multiple testing. Furthermore, ω-3 and ω-6 PUFA were not associated with WMH and PSMD. However, only 17 individuals showed lacunes, 7 individuals had MRI signs of infarction, and PSMD results were available from 73 participants.

## DISCUSSION

Our results support the view that the composition of free plasma PUFAs is linked to cognitive function and the preservation of brain volume in non-demented elderly individuals. In ASPS, higher plasma concentrations of free ω-6 PUFAs, and in particular linoleic acid, were significantly associated with lower executive function. In line with this clinical finding, we also saw that the volume of the frontal lobes was smaller in the presence of higher ω-6 PUFA concentrations. In contrast, higher ω-3 PUFA concentrations were not significantly related to cognitive functioning and the association with larger temporal lobe volumes did not withstand correction for multiple comparisons. We did not find an association of PUFAs and WMH, lacunes, infarctions or PSMD. Yet the number of patients with vascular lesions was small as was the number of study participants who underwent diffusion tensor imaging.

Previous studies have demonstrated a predictive role of PUFAs for cognitive decline and the risk of dementia [18, 19, 24]. Most of these studies reported significant associations between ω-3 PUFA and cognitive function. For example, in the longitudinal Three City Study with over 1200 participants, Thomas et al. found a 13% decrease in the risk of dementia per one SD higher plasma ω-3 PUFA concentration [18]. Moreover, ω-3 PUFAs were also associated with global cognition and memory. Similar results were obtained in community-dwelling elderly Japanese [19] and in patients with coronary artery disease [24]. In a supplementation study by Soininen et al. the administration of Fortasyn Connect, a combination of ω-3 PUFAs and multiple vitamins, slowed down the cognitive decline in subjects with prodromal AD [57, 58]. In contrast, other studies failed to replicate these findings in both demented and non-demented individuals [59, 60]. Also, in our cohort, ω-3 PUFA plasma concentrations were not associated with cognitive function. Previous literature reported conflicting results for ω-6 PUFAs as well. Our results corroborate studies from Australia and Japan that found a higher concentration of total ω-6 PUFAs and AA in patients with cognitive impairment and dementia than in controls [61–63]. Others found no such differences [26, 64]. In a meta-analysis of 10 cross-sectional and case-control studies, Lin et al. reported higher EPA, DHA and total ω-3 PUFA concentrations in non-demented controls than in dementia patients but the ω-3/ω-6 PUFA ratio did not significantly differ between these groups [27]. However, most of the studies included in this meta-analysis were rather small case-control studies that compared mixed cohorts of demented patients and non-demented controls. Furthermore, dementia and cognitive function were differently assessed hampering a direct comparison between studies. The ASPS study extends previous work as it included a large cohort of 391 non-demented individuals that underwent in-depth cognitive testing which captured different domains of cognitive function. Moreover, an extensive panel of 10 FA species was studied with a fully validated and quality-controlled GC-MS method. This method detects t-FFAs including FAs that are loosely bound to albumin and FAs that circulate freely in blood. Another strength of ASPS is the inclusion of volumetric MRI measurements. The association between higher ω-6 PUFA concentrations with smaller frontal lobe volume and executive dysfunction is biologically plausible, because the frontal cortex is traditionally considered the major brain structure involved in executive functions [65–68]. It is of note, however that this association was not particularly strong and lost significance after correction for multiple testing. Another finding of interest was the positive relationship between ω-3 PUFAs and temporal lobe volume. Although in our analysis the association lost significance after correction for multiple testing, several previous reports made a similar observation [18, 28]. In 467 non-demented participants of the Three City Study with >1 MRI scan over a median follow-up of 4.0 years, higher EPA + DHA concentrations which are major constituents of the ω-3 PUFA fraction, were significantly associated with less atrophy of the temporal lobes and slower cognitive decline over the observational period [18]. The authors calculated that per 1 SD increase in ω-3 PUFA concentration, the loss of mean medial temporal lobe volume decreased by 0.02 cm^3^/year. Another investigation of the same group mainly confirmed their previous results [28]. An *in vivo* study on monkeys is also in line with these epidemiological data as it showed that the lipid composition of the diet has a direct influence on the PUFA pattern in the temporal lobe [69]. Importantly, the impact of dietary lipid intake on the PUFA composition at brain tissue level differs between specific brain regions [70, 71]. This might explain our observation that differences in free plasma PUFA concentrations affect some, but not all, brain regions. WMH and PSMD were both unrelated to free plasma PUFA concentrations. This observation supports the concept that PUFAs influence brain function and volume primarily through a modulatory effect on cerebral lipid composition. However, the low number of cases in whom these and other structural parameters were available limits statistical power.

PUFAs may contribute to brain aging and cognitive decline in several ways. The balance between ω-3 and ω-6 PUFAs is essential for neural development during childhood and adolescence, and remains important throughout life for membrane fluidity, the prevention of inflammatory states, and cardiovascular health [72]. For example, the brains from AD patients exhibit reductions in DHA and the DHA derived mediator neuroprotectin D1 (NPD1), which protects against cell injury-induced oxidative stress [73]. NPD1 is not only anti-inflammatory and neuroprotective, but also harbors anti-amyloidogenic activity [74]. In mice, administration of a DHA-rich diet increased the concentration of brain-derived neurotrophic factor (BDNF), which promotes synaptic plasticity and improves learning and memory function [75]. Furthermore, in mice DHA protects against β-amyloid (Aβ) production and deposition, and cerebral amyloid angiopathy [75–77].

Our results suggest that the frontal and temporal lobe are particularly sensitive to imbalances of ω-3 and ω-6 free PUFAs. It is important to note that the observed effects of ω-3 and ω-6 PUFAs on cognition and brain structure are subtle and might thus be of limited clinical relevance. Longitudinal studies should clarify the prospective risk of PUFA imbalances for accelerated cognitive decline and dementia. Moreover, the impact of the truly unbound PUFA fraction on cognitive function and the risk of dementia needs to be further explored.
